# In Vitro Antioxidant, Antithrombotic and Anti-Inflammatory Activities of the Amphiphilic Bioactives Extracted from Avocado and Its By-Products

**DOI:** 10.3390/antiox14020146

**Published:** 2025-01-26

**Authors:** Anita Marra, Vasileios Manousakis, Nikolaos Koutis, Georgios Panagiotis Zervas, Anna Ofrydopoulou, Katie Shiels, Sushanta Kumar Saha, Alexandros Tsoupras

**Affiliations:** 1 Hephaestus Laboratory, School of Chemistry, Faculty of Sciences, Democritus University of Thrace, St Lukas, 65404 Kavala, Greece; anmarra@chem.duth.gr (A.M.); vamanvu@chem.duth.gr (V.M.); nikouti@chem.duth.gr (N.K.); geperva@chem.duth.gr (G.P.Z.); anofrid@chem.duth.gr (A.O.); 2 Shannon Applied Biotechnology Center, Technological University of the Shannon: Midlands Midwest, Moylish Park, V94 E8YF Limerick, Ireland; katie.shiels@tus.ie (K.S.); sushanta.saha@tus.ie (S.K.S.)

**Keywords:** avocado, by-products, antioxidant, antithrombotic, anti-inflammatory, polar lipids, phenolics, carotenoids, UFA

## Abstract

The antioxidant, antithrombotic and anti-inflammatory effects of the amphiphilic compounds extracted from both avocado juice and by-products, were evaluated. All extracts were assessed for their total phenolic content (TPC) and total carotenoid content (TCC), and for their antioxidant activities by DPPH, ABTS and FRAP assays as well as for their anti-inflammatory and antithrombotic potency in human platelets. The extracts rich in TAC (Total Amphiphilic Content) showed much higher content in phenolics and carotenoids from the extracts of total lipophilic content (TLC), which was reflected by the much stronger antioxidant capacities of TAC extracts. ATR-FTIR spectroscopy revealed the presence of not only phenolics and carotenoids, but also of bioactive polar lipids (PLs) in avocado TAC extracts, the LC-MS based structural analysis of which further revealed a fatty acid composition favourable for unsaturated fatty acids (UFAs) versus saturated ones (SFAs), including monounsaturated fatty acids (MUFAs) like the oleic acid (C18:1n9) and omega-3 (n3) polyunsaturated fatty acids (PUFAs) like the alpha linolenic acid (C18:3n3), with the subsequent anti-inflammatory low values of the n6/n3 PUFA ratio. The presence of such bioactive PLs that are rich in UFA within the TAC extracts of avocado juice and its by-products provide an explanation for the observed potent anti-inflammatory and antithrombotic activities of avocado TAC against thrombo-inflammatory mediators like platelet activating factor (PAF) and against standard platelet agonists like ADP, offering promise for such avocado TAC extracts, as ingredients in functional products for health/promoting applications either in cosmetics or in functional foods and nutraceuticals, or even drugs.

## 1. Introduction

Avocado, particularly valued for its nutritional richness, includes bioactive compounds located in the pulp, seed, and peel, solidifying its position as a functional food with extensive health benefits [1]. Often referred to as “vegetable butter”, due to its high unsaturated fat content, avocado also contains essential vitamins (notably C and D), minerals (such as copper), and phytochemicals like flavonoids, which contribute to its antioxidant and anti-inflammatory properties [2,3]. These bioactives are known to enhance immune function, mitigate inflammation, and reduce the risks associated with chronic diseases [4,5]. Its wealth of secondary metabolites and active molecules, including phenolics, tannins, carotenoids, and anthocyanins, further bolster avocado’s antioxidant profile, which highlights its potential role as a protective dietary component [3].

Researching the bioactive constituents of avocado has emerged as a promising area in the prevention and management of inflammation-related chronic diseases, including cardiovascular diseases, cancer, diabetes, and respiratory disorders. These conditions contribute significantly to global mortality, particularly in developed and developing nations, where they not only affect health, but also burden daily life and economic systems worldwide [6,7,8]. Risk factors associated with these diseases, both modifiable and non-modifiable, can foster persistent inflammation and oxidative stress, fuelling disease progression [9]. While medical treatments remain the primary approach, concerns over side effects, costs, and accessibility can limit their effectiveness [10]. Bioactive compounds in foods, and especially fruits, not limited to citrus fruits [11] but also in oily fruits like avocado, however, present a natural alternative for supporting health without adverse effects [12]. For those unable to maintain a daily intake of these bioactives through diet, the development of supplements and nutraceuticals derived from avocado provides a practical and accessible option [13,14,15].

In terms of specific health effects, research shows that avocado consumption supports cardiovascular health by enhancing blood lipid profiles, improving endothelial function, and reducing risk factors for heart disease [4,5]. Additionally, bioactive compounds found in avocado, such as carotenoids and xanthophylls (e.g., lutein and zeaxanthin), have demonstrated anticancer properties, particularly against tumours in the breast, larynx, and oral regions [1]. Avocado’s benefits also extend to diabetes management, with compounds that inhibit enzymes involved in glucose metabolism, potentially reducing rapid increases in blood sugar levels [16]. The evidence thus supports avocado’s multifunctional health effects, offering a dietary tool for reducing chronic disease risks through bioactive intake [17].

With over three million tons of avocados produced globally each year, where only the pulp is typically consumed, the seeds and peel often become waste, underscoring the need for sustainable approaches to avocado by-products [1,18,19]. A circular economy approach to these by-products aligns with UN and EU sustainability directives, highlighting their utility as cost-effective sources of bioactives that can be repurposed for health-promoting applications [13,14,15]. Rich in bioactives like monounsaturated fatty acids, tocopherols, polyphenols, and carotenoids, avocado by-products exhibit anti-inflammatory and antimicrobial effects, positioning them as valuable resources for pharmaceutical, nanotechnological, and tissue engineering applications [19,20,21]. Moreover, caffeoylquinic acid from avocado could enhance drug bioavailability, while polysaccharides in seeds and peels may be converted into biomaterials, such as polylactic acid (PLA), further supporting green technology and bioproduct development while lipid-soluble polyphenols that are primarily composed of caffeic acid derivatives such as caffeic acid ethylester are potent antioxidants known to inhibit cancer cell growth and lipid oxidation [19,20,22,23].

Consequently, the aim of the present study was to evaluate the physicochemical characteristics of the fruits, peel and pulp of *Persea americana* Mill. (avocado) of Cretan biological origin, with particular emphasis on the quantification of the content of lipophilic, amphiphilic bioactive constituents (i.e., carotenoids and phenolics, respectively) and the determination through biological platelet aggregometry assays of the different lipid extracts of avocado by-products, as well as the evaluation of their antioxidant and anti-inflammatory capacity, together with their structural elucidation.

## 2. Materials and Methods

### 2.1. Materials, Reagents and Instrumentation

Organic cultured Cretan Hass avocado fruits (three in total) of the Fuerte variety grown in Crete were the plant material from which the samples were prepared. Specifically, the fruits were sampled from orchards in the prefecture of Rethymnon: Koumpes area of the Municipality of Rethymnon. All reagents (Folin-Ciocalteu, Na_2_CO_3_, DPPH, ABTS), solvents (chloroform, methanol, petroleum ether, ethanol, n-octane and isopropanol), phenolic and lipid standards (Trolox, gallic acid, quercetin, catechin, soybean polar lipids and beta-carotene, respectively) were purchased from Sigma Aldrich. All UV-Vis spectroscopy analyses were performed on an LLG-uniSPEC 2 spectrophotometer and ATR-FTIR spectroscopy analyses on a Perkin Elmer Frontier ATR/FT-NIR/MIR spectrometer (Perkin Elmer, Waltham, MA, USA).

### 2.2. Extraction

Total lipids (TLs) were extracted from juices and by-products of three (n = 3) avocado samples of approximately 200 g each (208 g, 217 g, and 171 g) and further separated into extracts containing total lipophilic compounds (TLCs) and total amphiphilic compounds (TACs), based on modifications of the [24] extraction and [25] separation procedures, according to Tsoupras et al. [26]. Briefly, all fruits were pressed to produce avocado juice, and the by-products of this process were separately collected and extracts of the respective fruit juice and its by-products were obtained by mechanically homogenizing them in a blender in the presence of a single-phase system made by appropriate volumes of food-grade HPLC solvents (chloroform:methanol:water, 1:2:0.8, *v*/*v*/*v*). The obtained avocado TL extracts were filtered from the precipitated residues with 110 mm filter papers under vacuum conditions by pumping in a Buchner filtration apparatus. Then, appropriate volumes of chloroform and water were added in order to establish a biphasic system (chloroform:methanol:water, 1:1:0.9, *v*/*v*/*v*), in which the TLs are present in the lower organic (chloroform) phase. The latter was collected separately in round-bottom flasks and evaporated to dryness in a rotary flash evaporator at 37 °C under a vacuum between 700 and 50 mbar (Buchi Rotavaporator) and then re-dissolved in a 1/1 (*v*/*v*) chloroform/methanol solution. The samples were transferred to small pre-weighed glass vials, in which all remaining solvents were further evaporated under a stream of nitrogen. The obtained extracts were weighed and stored under nitrogen atmosphere at −20 °C (for a maximum of 6 weeks) for further analysis.

The obtained TL extracts of all samples were then further separated into their TAC and TLC fractions by counter-current distribution, based on a biphasic system of pre-equilibrated petroleum ether and 87% ethanol within separatory funnels, repeated several times until all TAC extracts were washed in the ethanol phase and TLC extracts in the petroleum ether phase, respectively. Both phases were collected in round-bottom flasks and evaporated with a rotary evaporator until dried, and the collected TLC and TAC extracts were transferred to small pre-weighed glass vials in small volumes of petroleum ether and chloroform:methanol 1:1 (*v*/*v*), respectively, and then further evaporated until they were dried and weighed. Each of the dried TAC samples was dissolved in 1 mL of ethanol, while each dry TLC sample was dissolved in 1 mL of octane. Then, 0.1 mL of each extract was aliquoted into individual test vials to conduct further analysis, and further evaporation of the small amounts of solvents used was performed to obtain dry TAC and TLC samples, which were then stored for further analysis under a nitrogen atmosphere at −20 °C.

### 2.3. Total Phenolic Content (TPC)

The TPC of all PL juice extracts and avocado by-products was evaluated using the Folin–Ciocalteu reagent, as previously described, by Tsoupras et al. [26]. More specifically, 1 mL of deionized water and 1 mL of Folin–Ciocalteu reagent were added to an aliquot of each extract. After 7 min, an additional 3 mL of Na_2_CO_3_ was transferred to each sample. The samples were then incubated in a dark place for 2 h. Between successive additions of reagents, and every 30 min during incubation, the solutions were stirred, using vortex. After the two-hour incubation of the samples, their absorbance at 765 nm was measured. The required concentration was measured on the basis of the standard gallic acid curve and the results obtained were expressed in mg gallic acid equivalents (GAE)/g DW.

### 2.4. Total Antioxidant Activity (TAA)

The evaluation of the antioxidant activity of the samples was carried out by three individual assays, namely the 1,1-diphenyl-2-picrylhydrazyl (DPPH) radical binding method, the cationic radical binding assay of bis-ammonium 2, 2′-azino-bis-(3-ethylbenzothiazolizolino-6-sulfonic acid) (ABTS) and via the trivalent iron reduction antioxidant power (FRAP) method, according to Tsoupras et al. [26].

Briefly, concerning the DPPH assay, 0.2 mL of ethanol, 0.8 mL of Tris-HCl buffer (pH 7.4) and 1 mL of DPPH solution were transferred to an aliquot of each extract. Between successive additions of the reagents, the samples were stirred using vortex, to mix the solutions. The samples were kept at room temperature for 30 min and immediately afterwards the absorbance at 517 nm was recorded.

The percentage of inhibition (%) was calculated from the following equation:

Inhibition (%) = (A1 − A2) × 100/A1,

where A1 is the absorbance of the control sample solution and A2 is the absorbance of the test sample solution.

The IC50, i.e., the concentration of each extract that has the ability to neutralise 50% of the DPPH radical, was then calculated. The DPPH radical scavenging activity of the sample was expressed as Trolox equivalent antioxidant capacity (TEAC). TEAC was calculated as follows:

TEAC = IC50 of Trolox (μg/L)/IC50 of the sample (μg/L).

For the ABTS assay, 2 mL of ABTS solution was transferred to an aliquot of each extract, followed by vortex stirring. The solutions were incubated in a dark place for 7 min, and immediately afterwards, their absorbance at 734 nm was measured. Trolox was used as a standard. The concentration of Trolox was chosen under the condition that the absorbance value ranged from 0.2 to 0.8, to draw a standard curve. 

The result was expressed as μmol TE/g DW, according to the formula

ABTS (μmol TE/g DW) = c × V × t/m,

where c is the Trolox concentration (µmol/mL) of the corresponding standard curve of the diluted sample, V is the volume of the sample (ml), t is the dilution factor and m is the dry weight of the sample (g).

For the FRAP assay, 3 mL of FRAP solution was transferred to an aliquot of each extract, followed by vortex stirring. The solutions were incubated in a dark place at 37 °C for 15 min and their absorbance at 593 nm was measured immediately afterwards. Trolox was used as a standard. The concentration of Trolox was chosen under the condition that the absorbance value ranged from 0.2 to 0.8, to draw a standard curve.

The result was expressed as μmol TE/g DW, according to the formula

FRAP (μmol TE/g DW) = c × V × t/m,

where c is the Trolox concentration (µmol/mL) of the corresponding standard curve of the diluted sample, V is the volume of the sample (ml), t is the dilution factor and m is the dry weight of the sample (g).

### 2.5. Total Carotenoid Content (TCC)

The quantification of the total carotenoid content (TCC) of each extract was carried out according to Tsoupras et al. [26]. Briefly, an aliquot of each extract was dissolved in 2 mL of octane and then its absorbance was measured at 450 nm. The required concentration was measured based on the standard β-carotene curve and the obtained results were expressed in mg of β-carotene equivalents (CE)/g of DW.

### 2.6. Evaluation of Fatty Acid Composition and Structural Elucidation of Polar Lipids in TAC Extracts by LC-MS

Liquid chromatography–mass spectrometry (LC-MS) was used to quantify the fatty acid composition of all extracts and to evaluate the overall structures of the PLs in the TAC extracts, as described by Tsoupras et al. [26]. Briefly, each extract was re-dissolved in 500 μL dichloromethane/methanol (1:2, *v*/*v*) and then centrifuged for 6 min at 13.000 rpm (Heraeus Biofuge Stratos, Fisher Scientific Ltd., Dublin, Ireland). The supernatant was then filtered through Sartorius Minisart Syringe Filters (0.2 µm, 15 mm PTFE, Sartorius, Göttingen, Germany). In order to obtain fatty acid profiles in these filtrates, 10 μL of each filtrate was injected into an HPLC system (Agilent 1260 series, Agilent Technologies Ireland Ltd., Little Island, Co., Cork, Ireland) equipped with a Q-TOF mass spectrometer (Agilent 6520) using electrospray ionization (ESI) as the source type. Separation of fatty acids was achieved using an Agilent C18 Poroshell 120 column (2.7 μm, 3.0 × 150 mm) with gradient elution, in which mobile phase A consisted of 2 mM ammonium acetate in water and mobile phase B consisted of 2 mM ammonium acetate in 95% acetonitrile. The mobile phase initially had a flow rate of 0.3 mL/min until 5 min elapsed, and was increased to 0.6 mL/min after 10 min and then maintained at this flow rate until the end of the run. The mass spectrometer scanned *m*/*z* values from 50 to 1100, while reference masses 1033.988 and 112.9855 were used to monitor the scan in negative ionization mode (Figure S1). The capillary voltage was 3500 V and the skimmer and fragmenter voltages were maintained at 65 V and 175 V, respectively. The drying gas flow, pressure and temperature of the nebulizer were set, respectively, to 5 L/min, 30 psi and 325 °C.

The validation of the LC-MS method was performed by comparing the specific accurate masses and retention time (RT) of various standards, such as saturated and unsaturated fatty acids, namely lauric (C12:0), myristic (C14:0), palmitic (C16:0), stearic (C18: 0), oleic (OA, C18:1n-9 cis), linoleic (LA, C18:2 n-6 cis), gamma-linolenic (GLA, C18:3 n-6), α-linolenic (ALA, C18:3 n-3), arachidonic (ARA, C20: 4 n-6), eicosapentaenoic acid (EPA, 20:5 n-3), docosahexaenoic acid (DPA, 22:5 n-3) and docosahexaenoic acid (DHA, 22:6 n-3) (Sigma, Dublin, Ireland). The peak area of each identified fatty acid was the average of triplicate sample injections, and their relative content was recorded according to their average peak area. Since the peak areas do not reflect the exact amounts of individual fatty acids, the relevant data should be read with caution.

The assignment of FFA and polar lipid (phospholipid and glycolipid) species was based upon a combination of survey, daughter, precursor and neutral loss scans, and the identity of the bioactive polar lipid molecules detected by the MS analysis at specific elution times (Figure S1), was further verified using the LIPID MAPS: Nature Lipidomics Gateway (www.lipidmaps.org, accessed on 25 January 2025), by using the lowest delta values combined with the results obtained from the LC-MS analysis on the fatty acid composition of the saponified PL, as previously described by Tsoupras et al. [26]. 

### 2.7. Evaluation of the Antiplatelet and Anti-Inflammatory Properties of Extracts by Cumulative Light Transmission Measurements

Bioassays to evaluate the antiplatelet and anti-inflammatory properties of all avocado extracts, as well as TL, TAC, and TLC extracts from all juice and by-product samples, were performed on human platelet-rich plasma (hPRP) preparations from healthy donors, on a Chrono-log 490 four-channel strobilometric platelet aggregometer (Havertown, PA, USA), connected to the accompanying AGGRO/LINK ®8 software package. All consumables for platelet aggregation were purchased from Chrono-log (Havertown, PA, USA). PAF, ADP and bovine serum albumin (BSA) standards were purchased from Sigma Aldrich (St. Liouis, MO, USA). The anti-inflammatory and antithrombotic activity of each sample was expressed as the mean IC50 (half-maximal inhibitory concentration) ± standard deviation (SD), quantified in mass (μg) of the bioactive lipid extract present in the aggregometer cuvette that can cause 50% inhibition of hPRP aggregation induced via the inflammatory and thrombotic mediator PAF or via the well-established standard platelet agonist ADP [26]. 

### 2.8. ATR-FTIR Analysis

In this method, a beam of infrared radiation passes through a high-refractive-index crystal, while a small portion of the radiation is absorbed by the sample in contact with the crystal. The absorption of the radiation causes vibrations of the chemical bonds in the sample, creating a spectrum which is recorded and analysed. This spectrum reveals information about the chemical composition of the sample, such as bond types, functional groups, and molecular structure. The ATR technique is particularly useful for the analysis of materials without special preparation, as it requires a minimal amount of sample and is non-destructive. More specifically, in our case, we used a small amount of avocado oil (i.e., extracts), which we dissolved in a small amount of isopropanol to obtain the spectra of our samples and standards, and we used the ATR-FTIR spectrophotometer technique (Perkin Elmer Frontier ATR/FT-NIR/MIR spectrometer) at a wavenumber range of 4000–600 cm^−1^, according to Vordos et al. [27]. In addition to the standards, each extract (sample to be analysed) was solubilized in a small amount of isopropanol, which served as the solvent. The sample was placed on the plate in an amount such that it covered the crystal, and then the tip was adjusted to touch the plate. As soon as these are in contact, a green line appears on the force gauge, and the force applied through the rotating tower is increased until the spectrum displayed on the work surface is stabilized. The spectrum obtained for each sample is analysed according to several standard samples for which the same procedure was previously followed. These standard samples are Quercetin, Catechin, Gallic Acid, and Beta-carotene, as well as polar lipids from soybeans. At the same time, the spectrum of isopropanol was also analysed to reduce the error in the study of the peaks due to solvent interference in the sample under study.

### 2.9. Statistical Analysis

The Kolmogorov–Smirnov method was used to test whether all parameters were normally distributed or not. Parameters that followed a normal distribution were compared using ANOVA (IC50 values against PAF and ADP), while all the other ones that did not follow a normal distribution were compared using non-parametric methods for comparing values using the Kruskal–Wallis method. A statistically significant difference between parameter values for each case was accepted when the *p* significance index was less than 0.05.

## 3. Results and Discussion

### 3.1. Yield of Extraction

The extraction efficiency for the recovered amounts of TL and its separated TAC and TLC fractions (expressed in g DW/100 g sample) from all avocado juices and by-products, respectively, are presented in Table 1.

All samples of both avocado juice and by-products contained high amounts of TAC, as the majority of the lipids were found to be neutral lipids (approximately 92%), which is consistent with the bibliography in extracts of avocado juice and by-products that were, however, obtained by conventional extraction methods [28,29]. The high yield of TACs from all parts of avocado underlines further their potential as a good anti-inflammatory source. Such experimental conditions applied in TAC extractions usually result in the obtainment of bioactive amphiphilic molecules, which are present as phenolics and polar lipids [26]. Thus, the higher the yield in such amphiphilic compounds, the better the anti-inflammatory potential for the derived extract.

After comparisons with other studies which equally calculated the extraction efficiency, it was observed that there were large variations in the values [20,30,31]. Variations in extraction yields may be due to different extraction methods, such as the use of different solvents or the application of different parameters, such as temperature and extraction time. In addition, the quality and origin of the plant material, as well as storage conditions, the process and storage of the avocado by-products, the inclusion or not of the avocado seed in the by-products and several other factors can significantly affect the extraction yield, leading to large variations between studies. 

Overall, avocado juice shows significantly higher concentrations in the categories of more TAC, more TLC, and TL, compared to avocado by-products. In particular, the concentration of the TL in avocado juice (mean 0.584, maximum 1.732, minimum 0.509) is much higher than that of by-products (mean 0.019, maximum 0.024, minimum 0.016). At the same time, the concentrations of TAC and TLC in the by-products are lower but also more stable, indicating greater homogeneity in the by-product samples than in the juice, which shows higher deviation values. 

### 3.2. Evaluation of Phenolic and Carotenoid Contents

#### 3.2.1. Evaluation of Carotenoid Content

The analysis for the presence of carotenoids in avocado juice and by-products, as shown in Table 2, reveals several differences in the concentrations of TAC, TLC, and TL extracts (Table 2). Carotenoid concentrations in TL and TAC are highest in both avocado juice and by-products, compared to their TLC. This high carotenoid content in TL and TAC suggests that carotenoids, which are important antioxidants, are mainly found in the total lipids and/or in amphiphilic phases of avocados. Carotenoids, such as beta-carotene, lutein, and zeaxanthin, are known for their potent antioxidant activity, which helps to protect cells from oxidative damage and maintain eye health. In addition, the presence of these compounds in avocado lipids is associated with cardiovascular benefits, as they can reduce LDL cholesterol oxidation and support cardiovascular health [32]. Moreover, the presence of such potent antioxidants in TAC can protect other amphiphilic bioactives that are susceptible to oxidation, such as PL rich in UFA, as observed in other oily food sources rich in carotenoid antioxidants and bioactive PL with UFA [26].

More specifically, as presented in Table 2, avocado juice has an increased carotenoid content in its TAC and thus in its TL, with values of approximately 58–60 mg CE/g DW. This suggests that carotenoids are highly associated with the amphiphilic compounds in the juice, which indicates that the majority of carotenoids found in TL were co-extracted in the TAC extracts due to the presence of amphiphilic compounds like PL in TAC, which are able to trap and retain carotenoids during extraction, instead of allowing them to be transferred to the TLC, which showed lower carotenoid content, with median values of approximately 1.5–4.1 mg CE/g DW.

Comparing the juice with the by-products, the median values of the carotenoid content of both TAC and TL seemed higher in the juice than in the by-products, but without any significant statistical difference. This indicates that by-products also contain considerable amounts of carotenoids in their TAC and TL extracts, while the carotenoid content of TLC is equally low in both juice and by-products. This suggests that TAC extracts from avocado by-products may be a good sustainable source of carotenoids that, as antioxidants, are suitable as functional ingredients in both cosmetic and nutritional applications, especially in terms of enhancing the antioxidant profile in such products [17].

#### 3.2.2. Evaluation of Phenolic Content

Analysis of total phenolic content in avocado by-products reveals that TL and TAC again possess significantly higher concentrations of phenolic compounds (with median values of approximately 400 mg to 800 mg GAE/g DW for TL and 300 mg to 700 mg GAE/g DW for TAC) compared to the TLC extracts from these sources (approximately 60–90 mg GAE/g DW) (Table 3). The results suggest that the high phenolic content observed in the TL of avocado by-products is mainly attributed to its amphiphilic components, as the more-lipophilic TLC extracts contain significantly lower amounts of phenolics. This may indicate the higher nutritional value and antioxidant capacity of avocado by-products’ TAC extracts compared to their TLC, which is important for their use industrially in nutritional or cosmetic applications with health-promoting properties, due to being highly bioactive with well-established antioxidant activities [17].

In avocado juice, TL showed high phenolic content, of approximately 505 mg GAE/g DW. This median value indicates the significant contribution of both TAC and TLC to the phenolic content of the TL of juice, since, differently from the avocado by-products, in the avocado juice the TLC contained similar phenolic content to that of the TAC extracts (*p* > 0.05 in these comparisons). The data show that phenolic compounds are almost equally distributed in the amphiphilic and lipophilic phases of the juice, which underlines the role of lipids in the biological activity of phenolics in avocados and avocado oils, regarding their antioxidant properties [17].

From the above, and by comparing the phenolic content of the juice with that of the by-products, it is observed that in the juice, similar to avocado oil, the phenolic content is partitioned between both the oily and more lipophilic compounds and the amphiphilic TAC, while in the by-products, the phenolic contents are mostly concentrated within the TAC extracts, which further suggest the significance of this sustainable source for antioxidant amphiphilic bioactives like avocado phenolics, which can be utilized in several health-related applications [17].

### 3.3. Total Antioxidant Activity (TAA) of Avocado TAC, TLC and TL Extracts

#### 3.3.1. ABTS Values of Antioxidant Capacity

Determination of antioxidant capacity of all extracts was performed by utilizing three different assays. The ABTS assay, in the ABTS+ cation, is produced by oxidation of ABTS and has a bright blue–green colour. Antioxidants reduce this cation, resulting in a discoloration of the solution. The reduction in absorbance is usually measured at a wavelength of 734 nm. The ability of the samples to neutralize the ABTS+ cation is compared with that of a standard antioxidant like Trolox (a synthetic analogue of vitamin E), and the results are expressed in μmol of Trolox equivalents (TEs). The ABTS assay is widely used to evaluate the antioxidant activity of foods, beverages and plant extracts [33,34]. The antioxidant capacity of all extracts from avocado juice and by-products, as measured by the ABTS method (μmol Trolox Equivalent/g dry weight), are presented in Table 4.

In avocado juice, its TAC extracts showed higher antioxidant activities than its TLC extracts (*p* < 0.05 in this comparison), with median ABTS values of approximately 20 μmol TE/g and 5 μmol TE/g, respectively, suggesting that the observed antioxidant capacity of the TL, with a median ABTS value of approximately 21 μmol TE/g, can mainly be attributed to the activity of their amphiphilic compounds. Similar outcomes are observed in the avocado by-products too, in which, again, their TAC extracts showed higher antioxidant activity than their TLC extracts (*p* < 0.05 in this comparison), with median ABTS values of approximately 13 μmol TE/g and 1.5 μmol TE/g, respectively, suggesting again that the observed antioxidant capacity of the TL, with a median ABTS value of approximately 14 μmol TE/g, can again be attributed to the activity of their amphiphilic compounds. 

The higher antioxidant activity of the TAC extracts in both the juice and the by-products can be attributed to their higher phenolic content, in synergism with their carotenoid content, while the lower, but considerable, antioxidant effects of their TLC extracts seem to mainly be associated with their carotenoid content. The presence of such bioactives in extracts from avocado juice and by-products further suggests beneficial roles for these extracts as functional ingredients against oxidative stress and associated health promotion [17]. 

#### 3.3.2. Antioxidant Capacity Measured by the DPPH Assay

The DPPH assay is based on the ability of antioxidants to neutralize DPPH free radicals, causing a change in the colour of the solution from purple to yellow, which can be measured spectrophotometrically. The greater the change in colour, the stronger the antioxidant activity of the substance under study. In the case of avocado, the DPPH assay provides important information on the ability of lipids, lipophilic and amphiphilic compounds to act as antioxidants, thus evaluating their potential in free-radical binding and protection against oxidative damage [33]. As mentioned before, both avocado juice and by-products are rich in bioactive phenolic and carotenoid compounds, which is reflected by the potent antioxidant capacity observed in the DPPH assay too, as presented in Table 5, with potential benefits against oxidative stress.

In both avocado juice and by-products, again, their TAC extracts showed higher antioxidant activities, with TEAC values of one order of magnitude higher than those observed for their TLC extracts in this assay too (*p* < 0.05 in this comparison), suggesting that the observed potent antioxidant capacity of the TL extracts in both the juice and in the by-products of avocado can mainly be attributed to the activity of their amphiphilic compounds. The higher antioxidant activity of the TAC extracts can again be attributed to their higher phenolic content, in synergism with their carotenoid content, while the lower, but considerable, antioxidant effects of the TLC extracts seem to mainly be derived fromtheir carotenoid content. The TEAC of avocado by-products was similar to that observed in the TAC of the juice, suggesting that not only avocado juice and oils, but also the TAC extracts of avocado by-products, can be exploited as bio-functional ingredients for products with enhanced antioxidant capacity, such as functional cosmetics or foods enriched with such amphiphilic natural antioxidants.

#### 3.3.3. Antioxidant Capacity Based on the FRAP Assay

The FRAP (Ferric Reducing Antioxidant Power) assay was utilized to evaluate the antioxidant capacity of TAC, TLC and TL extracts from both avocado juice and by-products, by quantifying the capacity of their antioxidant bioactives to recover ferric ions (Fe^3^⁺) in the divalent form (Fe^2^⁺). As mentioned before, both avocado juice and by-products are rich in bioactive phenolic and carotenoid compounds, which is reflected by the potent antioxidant capacity observed in the FRAP assay too, as presented in Table 6, with potential benefits against oxidative stress. In the FRAP analysis, the high antioxidant capacity of avocado is associated with the presence of mainly amphiphilic, but also lipophilic, antioxidants, which contribute to overall protection against oxidation in both food stuff and in human health.

In both avocado juice and by-products, again, their TAC extracts showed higher antioxidant activities than their TLC extracts in this assay too (*p* < 0.05 in this comparison), with median TE values of approximately 180 μmol TE/g and 490 μmol TE/g for the TAC extracts of both of juice and by-products, and 17 μmol TE/g and 25 μmol TE/g for the TLC extracts, respectively, suggesting that the observed potent antioxidant capacity of the TL extracts in both the juice and in the by-products of avocado can mainly be attributed to the activity of their amphiphilic compounds. The higher antioxidant activity of the TAC extracts can again be attributed to their higher phenolic content, in synergism with their carotenoid content, while the lower, but considerable, antioxidant effects of the TLC extracts seem to mainly be derived from their carotenoid content. 

In comparison to both the ABTS and DPPH assays that measure the free-radical scavenging activities, meaning the relative abilities of antioxidants to scavenge the ABTS and DPPH radicals in aqueous phases, instead, the FRAP assay measures the antioxidant potential in samples through the reduction of ferric iron (Fe^3+^) to ferrous iron (Fe^2+^) by antioxidants present in the samples, meaning it is a test reflecting the ferric-reducing ability of plasma, which is closer to the in vivo conditions, as previously mentioned [26,33]. Thus, the higher activity observed in the FRAP assay for the TAC extracts of both avocado juice and by-products compared to the low activities of their TLC extracts observed in the same assay, further reflects the abilities of the TAC extracts to show antioxidant effects in conditions that are closer to the in vivo situations like those for the FRAP assay (ferric-reducing ability of blood plasma), in comparison to the more in vitro-based assays of the ABTS and DPPH assays. Thus, the presence of both phenolic and carotenoid antioxidant bioactives in the TAC extracts from avocado juice and by-products further suggests beneficial roles for these extracts as functional ingredients against oxidative stress and associated health promotion [17].

### 3.4. ATR-FTIR Analysis of Avocado TAC Extracts

ATR-FTIR analysis facilitates an initial identification of the presence of specific bioactive compounds in an extract like the bioactive TAC extracts from avocado, which showed strong antioxidant effects. Through this technique, bioactive compounds like several phenolics and carotenoids, which are known for their antioxidant properties, as well as bioactive polar lipids (PLs) with potential anti-inflammatory and antithrombotic effects, can be detected. As presented in Table 7, ATR-FTIR analysis of the TAC extracts from both avocado juice and by-products, in comparison to fingerprint regions observed in known standards that were also assessed in the same conditions, showed that both TAC extracts indeed contain specific phenolic bioactives, with functional groups similar to those of highly bioactive flavonoids such as the highly bioactive flavonol, Quercetin, as well as in phenolic carboxyl groups of phenolic acids like the hydrolysable tannins (i.e., gallic acid), with similar peaks to the standards (quercetin and gallic acid), at ≈3300 cm^−1^ of phenolic O-H, ≈2950 cm^−1^ (C-H) and ≈1750–1700 cm^−1^ for phenolic -C=O). In addition, in both TAC extracts, the AFT-FTIR analysis also showed the presence of bioactive polar lipids, with similar peaks to the standard (soya-derived polar lipids) at ≈2922 cm^−1^, ≈2858 cm^−1^ and ≈1729 cm^−1^), and carotenoids with similar peaks to the standard (β-carotene) at ≈2927 cm^−1^ (C-H) and ≈1721 cm^−1^ (extended (-C=C-)).

More specifically, in the region around 3400 cm^−1^, a broad absorption is observed in both TAC extracts, which is typical of -OH (hydroxyl) groups. This region is associated with the presence of alcoholic groups like those in bioactive phenolic compounds found in avocado, and in carboxyl groups in some phenolic acids, which are potent antioxidants present in by-products of plant sources like avocado. The peaks around 3000 cm^−1^ are attributed to the vibrations of the C-H bonds in the methyl (-CH₃) or methylene (-CH_2_^−^) groups found usually in saturated acyl and alkyl chains of fatty acids of the PL contained in oily sources like avocado. A significant absorption was observed in the region between 1750 and 1700 cm^−1^, which is probably related to the vibration of the carbon–oxygen (C=O) double bonds found in carboxyl ester functional groups such as those in the esterified fatty acids of polar lipids present in the TAC extracts and in carboxyl groups of phenolic acids like gallic acid, but also in carbonyl functional groups, and especially in phenolic ketones or aldehydes like those present in flavonoids; these include the highly bioactive flavonol quercetin, as also observed in these standards (soy-derived polar lipids, gallic acids and quercetin), when assessed in the same experimental conditions. 

Interestingly, in the 1700–1550 cm^−1^ region, there are peaks (i.e., at 1650–1600 cm^−1^) associated with C=C and C-C vibrations of such bonds in resonance, such as those in phenolic rings of presumably phenolic bioactives like flavonoids, present in avocado TAC extracts, as also observed in the tested standards, the flavan-3-ol catechin, the flavonol quercetin, and in gallic acid, too. The C=C and C-C vibrations of such bonds in resonance detected in these peaks also indicate the presence of carotenoid bioactives, as also observed when assessing the standard β-carotene.

In the 1500–1000 cm^−1^ range, strong absorptions are attributed to C-O vibrations of ether and alcohol groups, characteristic of ester groups of esterified fatty acids in PL. Indeed, in avocado, polar lipids in which several UFA are esterified have also been previously detected [17]. This functional group (C-O) is also present in ether and ester groups of phenolic compounds like gallic acid and flavan-3-ols like catechin, while in the same range (1500–1000 cm^−1^) C-O-C vibrations are also detected like those present in alkyl PL and in flavan-3-ols like catechin Both these classes of bioactive molecules present potent anti-inflammatory and antithrombotic properties and cardioprotective actions, whi8le their presence has also been previously detected in avocado samples [17]. Finally, in the region around 900 cm^−1^, absorption associated with C-H or ring vibrations in aromatic compounds was also observed. These absorptions further indicate the presence of phenolic components in both TAC extracts, which are known for their antioxidant properties [17].

### 3.5. Anti-Inflammatory and Anti-Platelet Properties of Avocado Extracts

**Figure 1:**
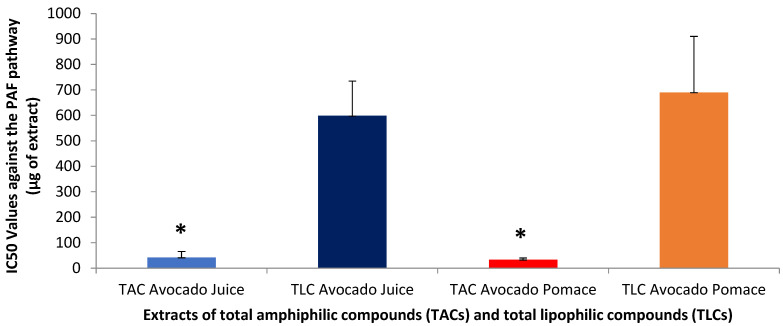
Inhibitory effects of total amphiphilic compounds (TACs) and total lipophilic compounds (TLCs) from avocado juice and by-products (pomace), against the PAF pathway. Results are expressed as IC50 values (half maximum inhibitory concentration), meaning the mass of the compound extract in μg present in the aggregometer cuvette containing 250 μL of human platelet-rich plasma (hPRP) that can cause 50% of inhibition of the PAF-induced inflammatory activation and aggregation of hPRP (the lower the IC50 value, the more potent the anti-inflammatory activity for an extract). * Indicates statistically significant difference, *p* < 0.05, between TAC and TLC in both the juice and the by-products (pomace).

The biological activities of all the extracts were evaluated by obtaining their putative anti-inflammatory and antiplatelet activities against the activation and accumulation of human platelets induced by the inflammatory and thrombotic mediator PAF, as well as by the classic platelet agonist ADP. Figure 1 illustrates the IC50 (half-inhibitory-concentration) values against the PAF pathway for all TAC and TLC extracts of avocado juice and its by-products.

The classical molecule of PAF (1-*O*-alkyl-2-acetyl-*sn*-glycero-3-phosphocholine) is a potent phospholipid mediator of inflammation, which, however, is involved in several inflammation-related pathologies and chronic disorders, including atherosclerosis, and the development of cardiovascular disease cancer. The primary role of PAF is to mediate cellular function and cell–cell interactions, which are critical in physiological processes, but also in pathological ones. PAF is involved in mediating physiological inflammatory responses, regulation of thrombotic responses, blood pressure, neurological function and reproduction, among other functions, through its cellular signalling via its G-protein coupled membrane receptor, PAF receptor (PAF-R). In both acute- and chronic-inflammatory states, PAF biosynthesis can be deregulated, leading to an increase in PAF levels, which can lead to systemic inflammation and associated disorders. In human platelets, PAF is a pro-inflammatory chemokine that induces thrombo-inflammatory signalling for shape change, aggregation and release of thrombotic granule contents through stimulation of the phosphatidylinositol cycle, intracellular Ca^2+^ mobilization and AA release [35].

With respect to the extracts derived from avocado juice, TAC showed a very low IC50 value, of only 42 μg of extract, indicating a strong inhibitory activity against PAF, since the lower the IC50 value for an extract, the more potent its inhibitory effect against a thrombo-inflammatory mediator. In contrast, the TLC of the juice has an order-of-magnitude higher IC50 value (approximately 599 μg of extract), indicating reduced efficacy of the TLC compared to the amphiphilic TAC compounds of avocado juice, against the PAF-pathway. 

Regarding avocado by-products, the TAC extracts, again, were the ones with an extremely low IC50 value, of only 33 μg of extract, and, thus, with a very potent anti-inflammatory anti-PAF effect, which was similar to that of the juice amphiphilic compounds (*p* > 0.05 for these comparisons), indicating that not only avocado juice and oil, but also the avocado by-products, can be a valuable and sustainable source of such amphiphilic bioactives, with potent anti-inflammatory properties. Similarly to the TLC from the juice, the TLC from avocado by-products showed an order-of-magnitude higher IC50 values (approximately at 690 μg of extract), confirming their lower efficacy compared to the TAC extracts (*p* < 0.05 for these comparisons).

These results show, for the first time, that amphiphilic compounds, both from the juice and by-products of avocado, have a stronger inhibitory effect against the PAF pathway compared to their lipophilic compounds. Amphiphilic compounds from avocado by-products show the highest efficacy, indicating that avocado by-products may be an important source of bioactive compounds. Meanwhile, it is important to note that the IC50 values found for avocado, especially for amphiphilic compounds, are relatively similar when compared to studies in amphiphilic extracts from other healthy fruits like apples and olives and their products (apple juice and olive oil) and by-products (apple pomace and olive pomace). This indicates that avocado is also a healthy fruit, rich in amphiphilic compounds, with strong anti-inflammatory and antiplatelet properties [36,37]. Moreover, the low IC50 values observed for the TAC of avocado by-products indicate a strong anti-PAF inhibitory activity, which may help to inhibit excessive platelet activation and thus reduce the risk of thrombosis. In addition, these substances may have anti-inflammatory activity, reducing the inflammatory response associated with PAF activity. In particular, the activity of avocado by-product extracts appears to be significant, which makes these by-products an interesting and potentially inexpensive source of bioactive ingredients for future pharmaceutical applications.

**Figure 2:**
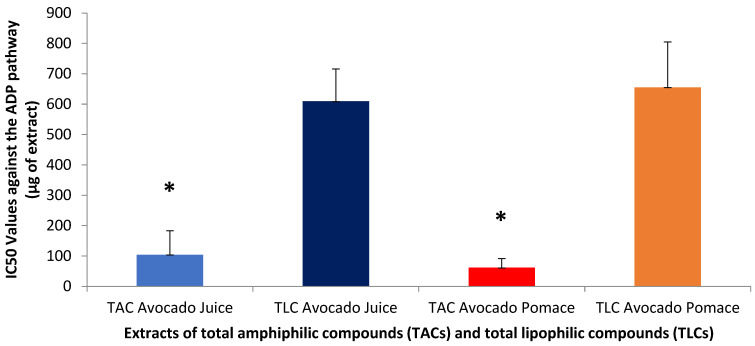
Anti-platelet effects of total amphiphilic compounds (TACs) and total lipophilic compounds (TLCs) from avocado juice and by-products (pomace) against the classic platelet agonist ADP. Results are expressed as IC50 values (half maximum inhibitory concentration), meaning the mass of the compound extract in μg present in the aggregometer cuvette containing 250 μL of human platelet-rich plasma (hPRP) that can cause 50% of inhibition of the ADP-induced thrombotic activation and aggregation of hPRP (the lower the IC50 value, the more potent the anti-inflammatory activity for an extract). * Indicates statistically significant difference (*p* < 0.05) between TAC and TLC in both the juice and the by-products (pomace).

With respect to the antiplatelet effects of TAC extracts from both the avocado juice and its by-products (pomace), Figure 2 shows the IC50 values of the TAC and TLC extracts from the respective avocado juice and by-products against the ADP-induced classic thrombotic pathway of hPRP aggregation. 

Similar to the observed outcomes for the PAF-pathway, in this assay, again, the TAC of both the avocado juice and the avocado by-products showed low IC50 values (of approximately 104 and 62 micrograms, respectively), indicating a high biological activity for this extract against this pathway, too. This means that a small amount of extract is required to inhibit the ADP pathway. On the other hand, TLC from either avocado juice or avocado by-products showed much higher IC50 values (approximately 610 and 655 micrograms, respectively), indicating lower activity of the TLC compared to TAC of the same sample against this pathway of thrombosis, too (*p* < 0.05 for all these comparisons). In summary, TAC extracts, both from avocado juice and avocado by-products, show stronger biological activity than TLC extracts, and, especially, the avocado by-product TAC shows the highest biological activity, making it more effective in inhibiting the ADP pathway, compared to the other samples.

ADP is a potent agonist of the platelet net receptors P2, P2Y1 and P2Y12 (formerly P2TAC), which are GPCRs, along with the ligand-induced P2 × 1 ion channel. Binding of ADP to these receptors is the first critical pathway of amplification in platelet activation. The ADP pathway is required for maximal platelet aggregation induced by other agonists. Platelets are activated upon binding to ADP, leading to shape change and release of granule contents, including ADP, which further alters platelet aggregation [35].

The results of the present study are in accordance also with previous analyses performed on the antiplatelet effect of avocado extracts and compounds. More specifically, Alejandro Rojas-García’s study showed that avocado may have an inhibitory effect on platelet activation. Although the study focused on the effect of adenosine, the results show that similar compounds or extracts, such as those contained in avocado, could reduce the activity of markers such as P-selectin and PAC-1, induced by ADP and collagen, enhancing its antiplatelet activity [38]. Moreover, the study by Dariana Graciela Rodriguez-Sanchez et al. showed that acetogenins isolated from avocado have significant antiplatelet activity, particularly in terms of inhibition of ADP-induced activation. Acetogenins such as Persenone-C showed strong inhibitory capacity, demonstrating that the structure of these compounds may contribute to the inhibition of the interaction of ADP with its receptors in platelets. Similar results were observed for other agonists such as collagen and arachidonic acid, although the action of acetogenins seems to be more pronounced against ADP. Compared to our results, the IC50 values for inhibition of platelet activation by ADP are lower for whole-avocado compound extracts (62–104 μg). compared to isolated acetogenins, where Persenone-C has an IC50 of about 3.42 mM. This indicates that avocado extracts are more effective than pure acetogenins in inhibiting ADP-induced platelet activation [39].

From the above, it can be summarized that the TAC from avocado juice and by-products show higher biological activity compared to extracts of TLC in both pathways, while extracts from avocado by-products seem to possess more potent biological activity than those from juice. Interestingly, by comparing the results for the TAC extracts between the two pathways assessed (ADP and PAF), the activity of avocado extracts against the PAF pathway was achieved by the presence of lower amounts of TAC extract compared to the IC50 values for inhibiting the ADP pathway, for both the avocado juice and its by-products. This means that avocado is more active and has better specificity against the PAF signalling pathway, probably due to an inhibitory (direct or indirect) effect against PAF binding to its receptor (anti-PAF inhibitory effect) by amphiphilic compounds with chemical affinity for the PAF-receptor and PAF-synthetic enzymes, such as PAF-like polar lipids rich in unsaturated fatty acids (UFAs). 

### 3.6. Fatty Acid Composition of the Bioactive TAC Extracts from Avocado Juice and Its By-Products

The fatty acid composition of the polar lipids (PLs) of avocado juice extracts and by-products was elucidated by LC-MS analysis, and the results are presented in Table 8. In the case of the results obtained after saponification, the PLs present in the TAC extracts from avocado juice showed higher amounts of UFA than their SFA content, while in the PL from avocado by-products their SFA content was higher than their UFA content. Within the UFA of the PL in TAC extracts from both sources, the monounsaturated fatty acids (MUFAs) were higher than the less abundant polyunsaturated fatty acids (PUFAs), which, however, were present in considerable amounts in both types of extracts.

Moreover, in all bioactive PL in the TAC extracts from both avocado juice and by-products, the most predominant fatty acids were the SFA palmitic acid (C16:0) and the MUFA oleic acid (OA, C18:1 c9 n9), followed by the SFA stearic acid (C18:0) and less, but considerable, amounts of the essential omega-6 (n6) PUFA linoleic acid (LA, C18:2 c9,12 n6) and the omega-3 (n3) PUFA, α-linolenic acid (ALA, C18:3 c9,12,15 n3), and much lower amounts of the MUFA palmitoleic acid (C16:1 c9). 

In the PL bioactives of the TAC extracts from avocado juice, the OA content was the most abundant one, almost twice as high as the SFA palmitic acid and stearic acid, which resulted in the MUFA content being equal to the SFA content in these PLs. In addition, the presence of the PUFA in these PL bioactives also contributes to the UFA of these PL bioactives, resulting in the UFA content being statistically significant higher than their SFA content. 

In contrast, in the PL bioactives of the TAC extracts from avocado by-products, the OA content was almost similar to that of the SFA palmitic acid or stearic acid, which resulted in the SFA content being much higher (*p* < 0.05) than the UFA content in these PLs, even though their PUFA content also contributed to their overall UFA content. Subsequently, the SFA content of the PL from the avocado by-products was higher than that of the PL in the avocado juice, and the MUFA content of the PL from the avocado by-products was lower than that of the PL in the avocado juice.

Nevertheless, the PL from the avocado by-products contained equal amounts of the highly bioactive PUFA content with that of the PL in avocado juice, suggesting a potential for these plant-derived by-products as sustainable sources of bioactive PL, rich in PUFA. Such rich-in-PUFA PL bioactives from several other natural sources, and especially from agro-food by-products, have shown strong anti-inflammatory and antithrombotic health-promoting effects against the thrombo-inflammatory mediators like PAF and thrombin, but also against established platelet agonists such as collagen and ADP, with subsequent preventative and therapeutic properties in several inflammation-related pathological situations. This further supports their valorisation from these bio-wastes as bioactive ingredients of innovative functional products like functional foods, supplements, nutraceuticals, cosmetics and pharmaceuticals [36,37]. 

Moreover, even though the relative content of n6 PUFA of the PLs present in the TAC extracts from both the avocado juice and its by-products was higher than their n3 PUFA content, the observed levels of the n-6/n-3 PUFA ratio in these bioactive PLs (approx. 3/1) were still found to be much lower than the 15/1. Taking into account the fact that healthy foods and diets contain usually beneficial low levels of this ratio, while the “Westernized” and processed unhealthy diets and foods are usually characterized by higher levels for this ratio, within the 15/1 limit [40], the results for the PUFA content with such a low n6/n3 PUFA ratio observed in the PL bioactives from both avocado juice and by-products further suggest a beneficial anti-inflammatory potential for these avocado-derived PL bioactives. This is because it has been suggested that, the lower the levels of this n6/n3 PUFA ratio in foods and diets, within the 15/1 limit, the better the preventive effects against inflammation and chronic disorders associated with platelet aggregation [40]. The above findings of the low levels of the n6/n3 PUFA ratios observed in PLs from all parts of avocados, not only provide a structural explanation for the observed potent anti-PAF and anti-ADP bioactivities for these PL bioactives assessed, but further support the anti-inflammatory and cardioprotective health-promoting properties of the rich-in-bioactive PL TAC extracts of avocados.

Additionally to the bioactivities observed in the rich-in-PUFA PL bioactives, the PUFA content of these PL has several beneficial biofunctions on its own, especially when released from the PL from cell membranes into cells, via specific cytoplasmic phospholipase A2 (PLA2) enzyme activities [36]. For example, PLA2-based release of an n-3 PUFA like ALA, from a bioactive PL present in cell membranes and/or lipoproteins after digestion, facilitates the production of anti-inflammatory eicosanoids intracellularly, which act antagonistically to other inflammatory and thrombotic eicosanoids (prostaglandins, leukotrienes, and thromboxanes), and which are usually produced via pro-inflammatory n-6 PUFAs like LA [36]. This further enhances the health benefits derived from the aforementioned n-3 PUFA, which can be released from avocado PL bioactives, while healthy dietary patterns based on these n-3 PUFAs have shown strong preventive benefits against various chronic disorders, such as in a Mediterranean diet enriched in ALA for the secondary prevention of coronary heart disease [40,41,42,43]. In the results of avocado extracts, each sample showed low, but considerably significant, levels of ALA n3PUFA, and high levels of the OA MUFA, which can also be considered beneficial against thrombo-inflammatory mediators and associated diseases [40,41,42,43]. More specifically, as mentioned before such bioactive PLs from fruit by-products rich in either the n3 PUFA ALA or the MUFA OA have been associated with potent anti-PAF and anti-ADP health-promoting properties [40,41,42,43]. This further suggests that it is not only the fatty acid content that provides bio-functionality to the bioactive PL extracted from avocados rather than from their overall structures. It appears that the co-presence of such bioactive polar lipids (glycolipids and phospholipids) in the avocado-derived TAC extracts, present in these extracts of potent antioxidants like the amphiphilic phenolic compounds and carotenoids of low molecular weight, not only further contribute to the overall anti-inflammatory activity for these TAC extracts against both the inflammatory and thrombotic pathways of PAF and ADP, but also provide an antioxidant protection to the susceptible-to-oxidation PUFA and MUFA contents of these PL bioactives in these TAC extracts, from both the avocado juice and avocado by-products.

### 3.7. Structural Elucidation of the Main PL Bioactives Present in the TAC Extracts of Avocado Pulp and By-Products Assessed

Avocado-derived TAC extracts from juice and its by-products were further analysed with LC-MS (Liquid Chromatography–Mass Spectrometry) for the identification of molecular species of the bioactive polar lipids of interest, which resulted in various polar lipid classes and molecular species being successfully identified, as shown in Table 9. 

With respect to the LC-MS structural analysis of PL in the TAC extracts from both avocado juice and by-products, survey scans in the negative ion mode between 600 and 1000 *m*/*z* demonstrated that the main glycolipid classes identified were sphingosine-based ones, specifically several molecular species of Hexosylceramides (HexCer), while the main phospholipid classes identified were glycerol-based ones (GP, glycerolphospholipids), such as several molecular species of phosphatidylcholines (PCs), phosphatidylethanolamines (PEs), phosphatidic acid (PA), phosphatidylinositols (PIs) and phosphatidylglycerols (PGs). Moreover, in all PL classes, both diacyl PL and alkyl-acyl PL of either glycerol-based phospholipids (PC, PE, PI, PG and PA) or sphingosine-based glycolipids (mainly the HexCer class) were detected. 

Similarly to what was observed by the quantification of the fatty acid composition of avocado PL, most of these PL bioactives were found to mainly contain mainly the MUFA oleic acid (C18:1 c9), followed by the palmitic (C16:0) and stearic (C18:0) SFA, but also the n6 PUFA LA and the n3 PUFA ALA, with the SFA tending to be present in the *sn*-1 position, while the UFA (either MUFA or PUFA) was commonly present at the *sn*-2 position of these PLs. This positional specificity strongly affects the biochemical properties and functions of PLs, including membrane fluidity and enzyme interactions [44], such as the aforementioned one with the PLA2, which provide anti-inflammatory intracellular signalling [35]. The resulting data were in accordance with previous works that structurally eluded the lipidomics of avocado, including its PL classes [29,45].

More specifically, in the PC and PE, which are the main classes of interest in the avocado PL, both alkyl-acyl and/or di-acyl PCs and PEs were identified, and especially molecules that seem to contain MUFA or PUFA in the *sn*2 position of their glycerol backbones. Such PLs have shown potent anti-PAF anti-inflammatory actions, since they can act as potent PAF antagonists or even agonists with lower activities, by blocking the binding of PAF to its specific receptor (PAF-r, PAF-receptor) and thus preventing the PAF-induced activation of several cells, including platelets, and subsequently the PAF/PAF-R-related thrombo-inflammatory cascades [36,37]. In platelets, such an antagonistic/agonistic inhibition of PAF-action by these PL bioactives reduces the PAF-induced platelet activation and aggregation, with subsequent preventative and therapeutic health-promoting effects against cardiovascular diseases and tumour metastatic procedures [35]. 

Also, after digestion, some portion of these dietary PL bioactives can integrate into lipoproteins and cell membranes of several cells, which can also beneficially influence their response to inflammation by activating processes resulting in increased HDL levels and functionality [46], and also cellular-lipid signalling pathways related to PAF activity, offering protective effects against thrombo-inflammatory manifestations involved in several diseases [35]. 

In addition, other studies support the fact that PGs and PIs, such as those identified in our study, are involved in inflammatory pathways, and could showcase important anti-inflammatory responses, whereas HexCers, such as those identified in our study, are involved in signal transduction, neuronal differentiation, synaptogenesis, and many other cellular processes in the brain, with proposed neuroprotective effects [47,48,49,50]. Moreover, the PAs identified are important phospholipids involved in the regulation of a number of key cellular processes, as important signalling molecules that activate the mechanistic target of rapamycin complex 1, a critical regulator of cell growth, metabolism, and protein synthesis [51].

## 4. Conclusions

Within the present study, it was found for the first time that extracts from both avocado juice and its by-products contain substantial amounts of amphiphilic bioactives, such as the rich-in-MUFA-and-PUFA bioactive PL, with potent anti-inflammatory and antithrombotic properties, as well as potent antioxidant phenolics and carotenoids. Thus, this study also includes novel data demonstrating that the avocado-derived TAC extracts exhibit potent antioxidant and anti-inflammatory activity, particularly against oxidative stress and PAF-related thrombo-inflammatory signalling, as well as substantial antiplatelet activity against signalling of standard platelet agonists like ADP, marking a significant advancement over previously published findings. Structural elucidation revealed a favourable UFA content in the PL bioactives from both avocado juice and by-products, bearing mainly MUFA and PUFA at the sn-2 position of their structures, and with favourable n6/n3 PUFA ratios that further support their anti-inflammatory potential. The co-presence of these PL bioactives with phenolic and carotenoid bioactives with potent antioxidant activities not only promotes a favourable inhibition of oxidative stress, too, but also an antioxidant protection of the susceptible-to-oxidation PUFA and MUFA content of the PL bioactives, as reflected by the high activities observed in both the antioxidant assays and the thrombo-inflammatory models. 

Overall, the findings confirm the potent anti-inflammatory, antioxidant and antiplatelet properties of TAC extracts from avocado, providing a basis for future applications in cosmetics and functional foods. More particularly, the antioxidant and anti-inflammatory properties of the fruit could be used in cosmetics applications for anti-aging skin protection against skin inflammation and UV-induced oxidative stress and aging or for post-sunburn pain-relief applications, while the potent inhibitory effects of avocado TAC extracts against thrombo-inflammatory mediators and their relevant pathways, which are involved in atherosclerosis and cancer development and progression, further suggests that these extracts could be utilized as bioactive ingredients in functional foods, nutraceuticals, or even drugs against CVD and cancer.

Moreover, the observed benefits in the TAC extracts from avocado by-products provide significant data for their valorisation as a new sustainable source of bioactive ingredients for such functional products with nutraceutical, cosmeceutical and pharmaceutical health-promoting applications against several inflammation- and oxidative stress-related manifestations, in a circular economy design; however, full utilization of such by-products requires further research and improvements in green extraction procedures with high recovery yields and activities.

## Figures and Tables

**Table 1:** Extraction yield for total lipids (TLs), lipophilic components (TLCs) and amphiphilic components (TACs).

Samples	TAC			TLC			TL		
	Median	Max	Min	Median	Max	Min	Median	Max	Min
Avocado juice	0.498	1.658	0.450	0.074	0.086	0.059	0.584	1.732	0.509
Avocado by-products	0.017	0.021	0.012	0.003	0.005	0.002	0.019	0.024	0.016

The results are expressed in g/100 g sample (median, max, min value, n = 3) for the three avocado samples assessed. Abbreviations: TAC = extracts of total amphiphilic compounds, TLC = extracts of total lipophilic compounds, TL = extracts of total lipids.

**Table 2:** Comparison of total carotenoid content for total amphiphilic compounds (TACs), total lipids (TLs), and total lipophilic compounds (TLCs) in avocado juice and by-products.

Samples	TAC			TLC			TL		
	Median	Max	Min	Median	Max	Min	Median	Max	Min
Avocado juice	57.90	58.01	25.83	3.14	4.19	1.46	59.47	4.19	30.02
Avocado by-products	27.26	89.07	12.11	1.47	3.90	1.21	28.46	92.97	13.58

Results are expressed as mg carotenoid equivalent (CE)/g DW, (median, max, min).

**Table 3:** Comparison of total phenolic content in avocado juice and by-products, for total amphiphilic compounds (TACs), total lipophilic compounds (TLCs), and total lipids (TLs).

Samples	TAC			TLC			TL		
	Median	Max	Min	Median	Max	Min	Median	Max	Min
Avocado juice	243.43	367.22	241.46	137.78	728.22	11.01	505.00	971.65	252.47
Avocado by-products	381.43	703.48	285.96	66.79	89.74	58.77	440.20	770.28	375.70

Results are expressed as mg gallic acid equivalent (GAE)/g DW, (median, max, min).

**Table 4:** Comparison of antioxidant capacity, for total lipids (TLs), total amphiphilic compounds (TACs), and total lipophilic compounds (TLCs) in avocado juice and its by-products.

Samples	TAC			TLC			TL		
	Median	Max	Min	Median	Max	Min	Median	Max	Min
Avocado juice	19.95 *	86.32	8.23	4.49	7.16	0.87	20.82	93.48	12.72
Avocado by-products	12.79 *	25.08	6.96	1.57	1.76	1.32	14.11	26.84	8.53

Results are expressed as ABTS values (μmol Trolox equivalent/g dry weight), * indicates statistically significant differences between TAC and TLC extracts.

**Table 5:** Comparison of Trolox equivalent antioxidant capacity (TEAC), for total lipids (TLs), total amphiphilic compounds (TACs), and total lipophilic compounds (TLCs) in avocado by-products.

Samples	TAC			TLC			TL		
	Median	Max	Min	Median	Max	Min	Median	Max	Min
Avocado juice	0.0610 *	0.0720	0.0154	0.0044	0.0010	0.0063	0.0547	0.0110	0.0710
Avocado by-products	0.0669 *	0.1088	0.0247	0.0002	0.0002	0.0002	0.0670	0.1089	0.0249

As measured by the DPPH assay (expressed as TEAC values), * indicates statistically significant differences between TAC and TLC extracts.

**Table 6:** Comparison of antioxidant capacity for total lipids (TLs), total amphiphilic compounds (TACs), and total lipophilic compounds (TLCs) in avocado by-products.

Samples	TAC			TLC			TL		
	Median	Max	Min	Median	Max	Min	Median	Max	Min
Avocado juice	179.88 *	402.39	35.65	16.92	44.19	4.92	224.07	419.31	40.57
Avocado by-products	494.46 *	904.34	265.36	25.34	49.36	12.02	482.44	929.68	314.72

As measured by the FRAP assay (μmol TE/g DW), * indicates statistically significant differences between TAC and TLC extracts.

**Table 7:** Characteristic infrared (IR) absorption peaks for the TAC extracts of avocado by-products and avocado juice, with corresponding functional group assignments.

Peak (cm^−1^)	TAC of Avocado Juice	TAC of Avocado By-Products	Bond/Functional-Group Correlation
3400–3300	+ *	+	O-H (hydroxyl) bonds, characteristic of this functional group in phenolic compounds
3000–2900	+	+	C-H (alkyl), typically from fatty acids and aliphatic compounds like those present in polar lipids (PLs)
1750–1700	+	+	C=O (carbonyl), characteristic of esters in polar lipids, but also in phenolic C=O (similar to quercetin) and COOH (similar to gallic acid)
1650–1600	+	+	C=C (double bonds in aromatic compounds, unsaturated fatty acids and carotenoids)
1450–1400	+	+	C-H (alkyl), often from aliphatic hydrocarbons such as those observed in saturated fatty acids in PL
1250–1200	+	+	C-O (ether and alcohol groups), indicative of ester groups in PL and ether and ester groups in phenolic compounds like gallic acid and flavan-3-ols like catechin
1050–1000	+	+	C-O-C (ether group) like those present in alkyl PL and in flavan-3-ols like catechin

* Indicates detected peak from the analysis of this extract.

**Table 8:** Fatty acid profile of the PL present in TAC extracts from both avocado juice and by-products after saponification, expressed for each fatty acid (FA) as percentage composition of total fatty acids in each sample evaluated (mean ± standard deviation (SD), n = 3).

Fatty Acids (Empirical Name)	Type of Fatty Acid (Carbon Atoms, Double Bonds, and Their Positions)	Avocado Juice	Avocado by-Products
Caprylic	C8:0	0.08 ± 0.02	0.12 ± 0.02
Pelargonic	C9:0	0.10 ± 0.02	0.2 ± 0.04
Lauric	C12:0	0.09 ± 0.00	0.33 ± 0.01
Tridecylic	C13:0	0.19 ± 0.03	0.35 ± 0.03
Myristic	C14:0	0.53 ± 0.03	0.85 ± 0.04
Pentadecylic	C15:0	0.21 ± 0.03	ND
Palmitic	C16:0	22.80 ± 1.23	30.61 ± 0.78
Palmitoleic	C16:1 c9 (n7 MUFA)	1.58 ± 0.08	1.39 ± 0.19
Margaric	C17:0	0.41 ± 0.02	ND
Stearic	C18:0	18.05 ± 2.11	29.42 ± 1.68
Oleic (OA)	C18:1 c9 (n9 MUFA)	41.34 ± 0.99	26.13 ± 0.54
Linoleic (LA)	C18:2 c9,12 (n6 PUFA)	11.10 ± 0.08	8.12 ± 0.09
Alpha linolenic (ALA)	C18:3 c9,12,15 (n3 PUFA)	3.43 ± 0.10	2.49 ± 0.11
Ardenic	C22:4 c7,10,13,16 (n6 PUFA)	0.08 ± 0.03	ND
SFA		42.47 ± 0.98	61.88 ± 0.9
UFA		57.53 ± 0.98 *	38.12 ± 0.9 *
MUFA		42.91 ± 0.99 **	27.52 ± 0.7 **
PUFA		14.61 ± 0.05	10.60 ± 0.2
n3 PUFA		3.43 ± 0.10	2.49 ± 0.1
n6 PUFA		11.18 ± 0.06	8.12 ± 0.0
n6/n3		3.26 ± 0.11	3.27 ± 0.1

Abbreviations: OA = oleic acid; LA = linoleic acid; ALA = alpha-linolenic acid; n3 = omega-3; n6 = omega-6; n9 = omega-9; SFA = saturated fatty acid; MUFA = monounsaturated fatty acid; PUFA = polyunsaturated fatty acid; ND = undetectable (usually defined as fatty acids detected with a contribution of less than 0.005% to the total fatty acid content). * Indicates statistically significant difference (*p* < 0.05) of SFA compared to UFA. ** Indicates statistically significant difference (*p* < 0.05) of MUFA compared to PUFA.

**Table 9:** Representative molecular species of the main classes of the polar lipid bioactives detected in the TAC extracts of avocado juice and by-products by LC-MS analysis.

	TAC Extracts from Avocado Juice				TAC Extracts from Avocado by-Products			
Main Classes of PL	Elution Time (min)	Mr	Representative Molecular Species	Proposed Structures	Elution Time (min)	Mr	Representative Molecular Species	Proposed Structures
PC	6.5–7.5	770.8636	PC 36:2	[i.e., PC 18:1/18:1 or PC 18:0/18:2]	2.5–3	786.8408	PC 36:2;O	[i.e., PC 18:1/18:1;O or PC 18:0/18:2;O]
	6.5–7.5	770.8636	PC O-36:3;O	[i.e., PC O-18:1/18:2;O or PC O-18:0/18:3;O]	7.5–10	770.8638	PC 36:2	[i.e., PC 18:1/18:1 or PC 18:0/18:2]
	6.5–7.5	786.8542	PC 36:2;O	[i.e., PC 18:1/18:1;O or PC 18:0/18:2;O]	7.5–10	770.8638	PC O-36:3;O	[i.e., PC O-18:0/18:3;O or PC O-18:1/18:2;O]
PE	10–10.5	714.4146	PE 34:2	[i.e., PE 16:1/18:1 or PE 16:0/18:2]	10–10.5	734.3890	PE 36:6	[i.e., PE 18:3/18:3]
	10–10.5	714.4146	PE O-34:3;O	[i.e., PE O-16:0/18:3;O or PE O-16:1/18:2;O]	10–10.5	714.4065	PE 34:2	[i.e., PE 16:0/18:2 or PE 16:1/18:1]
	10–10.5	744.4252	PE 36:1	[i.e., PE 18:0/18:1]	10–10.5	714.4065	PE O-34:3;O	[i.e., PE O-16:0/18:3;O or PE O-16:1/18:2;O]
	10–10.5	698.4195	PE O-34:3	[i.e., PE O-16:0/18:3 or PE O-16:1/18:2]	10–10.5	744.4155	PE 36:1	[i.e., PE 18:0/18:1]
					10–10.5	698.4123	PE O-34:3	[i.e., PE O-16:0/18:3 or PE O-16:1/18:2]
PG	12–12.5	745.5059	PG 34:2	[i.e., PG 16:1/18:1 or PG 16:0/18:2 ]	12–13	745.5015	PG 34:2	[i.e., PG 16:0/18:2 or PG16:1/18:1]
	12–12.5	745.5059	PG O-34:3;O	[i.e., PG O-16:0/18:3;O or PG O-16:1/18:2;O]	12–13	745.5015	PG O-34:3;O	[i.e., PG O-16:0/18:3;O or PG O-16:1/18:2;O]
PI	12–12.5	837.5688	PI 34:0	[i.e., PI 16:0/18:0]	12–13	821.4921	PI 32:2;O	[i.e., PI 16:1/16:1;O]
	12–12.5	837.5688	PI O-34:1;O	[i.e., PI O-18:0/16:1;O or PI O-16:0/18:1;O]	12–13	843.5332	PI O-36:4	[i.e., PI O-18:1/18:3 or PI O-18:2/18:2]
	12–12.5	843.5303	PI O-36:4	[i.e., PI O-18:1/18:3 or PI O-18:2/18:2]				
PA					13.917	677.5396	PA O-34:0;O	[i.e., PA O-16:0/18:0;O]
					13.917	677.5396	PA O-36:6	[i.e., PA O-18:3/18:3]
HexCer	6.5–10	792.8578	HexCer 36:0;O6	[i.e., HexCer 18:0/18:0;O6]	6.5–10	792.9	HexCer 36:0;O6	[i.e., HexCer 18:0/18:0;O6]
	10–10.5	744.4252	HexCer 34:2;O5	[i.e., HexCer 16:1/18:1;O5 or HexCer 16:0/18:2;O5]	10–10.5	744.4155	HexCer 34:2;O5	[i.e., HexCer 16:0/18:2;O5 or HexCer 16:1/18:1;O5]

Abbreviations: PL = polar lipid; PC = phosphatidylcholine; PE = phosphatidylethanolamine; PA = phosphatidic acid; PI = phosphoinositol; PG = phosphoglycerol; HexCer = sphingosine-based hexosylceramides (glycolipids).

## Data Availability

All data are contained within the article. Any further information concerning raw data (i.e., FT-IR Spectra, LC-MS chromatograms and Spectra) can be provided by the Authors, upon request.

## Supplementary Materials

The following supporting information can be downloaded at: https://www.mdpi.com/article/10.3390/antiox14020146/s1. Figure S1: Representative Chromatogram of avocado extract with some ESI-MS analysis, as observed and obtained during the Analysis.
