# Supplementary material for: In Vitro Antioxidant, Antithrombotic and Anti-Inflammatory Activities of the Amphiphilic Bioactives Extracted from Avocado and Its By-Products
Source: Antioxidants (Basel). 2025 Jan 26;14(2):146. doi: 10.3390/antiox14020146 (PMC11851995; doi:10.3390/antiox14020146)

antioxidants

MDPI

Supplementary Figure S1: Representative Chromatogram of avocado extract with some ESI-MS analysis, as observed and obtained during the Analysis

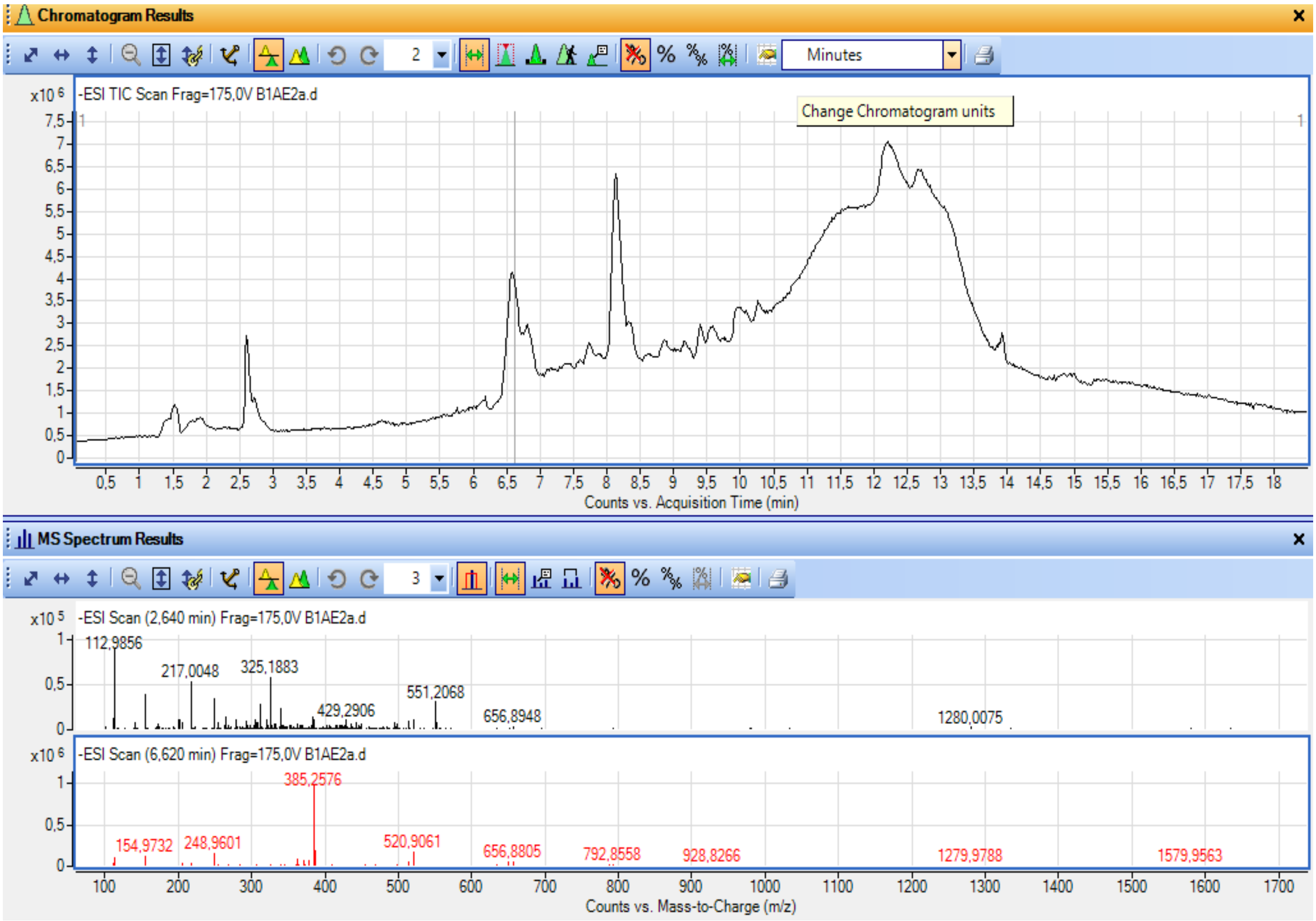

Supplement: Supplementary file 1 [file antioxidants-14-00146-s001.zip › antioxidants-3382266-supplementary.pdf]
